# Organosulfur and Organoselenium Chemistry

**DOI:** 10.3390/molecules29235523

**Published:** 2024-11-22

**Authors:** Ming Wang

**Affiliations:** School of Chemistry and Molecular Engineering, East China Normal University, Shanghai 200062, China; wangming@chem.ecnu.edu.cn

Organosulfur- and organoselenium-containing compounds play a crucial role in organic synthesis. These sulfur-containing molecules are widely applied in pharmaceuticals [1,2], materials [3,4,5], natural products [6], and food [7,8]. They are also useful building blocks in organic synthesis. Additionally, sulfur and selenium show divergent functions and potencies in different oxidative states, leading to the rich chemistry of their transformations. This *Molecules* Special Issue introduces organosulfur and organoselenium chemistry in fields such as organic synthesis, material, chemical biology, and computational chemistry, and contains eleven articles and four reviews. We collected excellent contributions from scientists across these different research areas, demonstrating the global reach of organosulfur and organoselenium chemistry; the authors contributing to this Special Issue hail from China, Canada, France, Germany, America, Poland, Japan, the Czech Republic, and Croatia. Six articles and three reviews reported on organosulfur chemistry, while five articles and one review reported on organoselenium chemistry.

The contributions related to organosulfur chemistry are summarized briefly below. Madec’s article (contribution 1) explored tetrahydro-4*H*-thiopyran-4-one 1-oxide and sulfinyl-di-*tert*-butylpropionate as sulfenate sources for the preparation of symmetrical biarylsulfoxides via pallado-catalyzed cross-coupling. Moreover, they discovered a thermally activated delayed fluorescence phenomenon on biarylsulfoxide products via their photophysical studies. Bi et al. (contribution 2) developed a reaction of acetone and thiols promoted by methanesulfonic anhydride/sulfuric acid for the synthesis of thioacetals/thioketals and β-sulfanyl ketones in excellent yields. Their strategy can furnish thioacetals/thioketals and thia-Michael addition products in a controllable manner. Smith’s group (contribution 3) explored five durable composites via a simple one-pot reaction of lignin oil and S_8_. These sulfur-containing composites were remelted and reshaped several times without losing mechanical strength, and displayed compressive strengths and flexural strengths exceeding that of ordinary Portland cement. Żurawiński et al. (contribution 4) reported the effect of sulfur oxidation on the structures and photophysical properties of [1]benzothieno[3,2-b][1]benzothiophene (BTBT) derivatives. Their studies found that the process of sulfur oxidation can change the crystal packing, thermal stability, and photophysical properties of BTBT derivatives. Wang et al. (contribution 5) developed a BF_3_·OEt_2_-mediated reaction of alkynes and sodium sulfonates for the preparation of β-keto sulfones in good yields. Their reaction was metal-free, performed under mild conditions and showed good functional group compatibility. Bi et al. (contribution 6) reported the FeCl_3_-catalyzed sulfonylmethylation of imidazo[1,2-α]pyridines, *N*,*N*-dimethylacetamide (DMA) and sodium sulfonates in H_2_O conditions (DMA:H_2_O = 2:1). Diverse 3-(sulfonylmethyl)imidazo[1,2-α]pyridines were furnished in good yields and showed excellent functional group compatibility. Their mechanism studies found that the current reaction may undergo an oxidation addition step, with DMA as the carbon source. The review by Zhao et al. (contribution 7) summarized the representative examples of desulfonylative reactions of inactive C(*sp*^2^)–SO_2_ bonds in (hetero)arylsulfones and their mechanistic insights into these accomplishments. Their review includes two parts: transition metal-catalyzed desulfonylative reactions and photo-/electrocatalytic radical desulfonylative reactions. The review of Krupka et al. (contribution 8) discussed the synthesis of thionated perylenediimides (PDIs) by using Curphey’s reagent (the association of phosphorus pentasulfide with hexamethyldisiloxane) to replace Lawesson’s reagent. This review demonstrated that Curphey’s reagent displays higher reactivity compared with Lawesson’s reagent for the synthesis of multi-thionated PDIs. Queneau’s review (contribution 9) summarized the strategies used for synthesizing thioamides using different sulfur sources over the past decade. Elemental sulfur, as one of the most practical and clean reagents used for the synthesis of thioamides, was highlighted in this review.

The contributions related to organoselenium chemistry are summarized below. Back et al. (contribution 10) performed a computational study on a variety of hetero-selenoxide *syn* elimination reactions, with the heteroatoms including O, N, and S. They found that selenoxides usually have lower activation energies, while the corresponding selenones exhibit higher activation energies. Hilt et al. (contribution 11) developed an electrochemical cross-electrophile coupling reaction by using aryl iodides and diselanes as electrophiles under the conditions of a Ni(acac)_2_ catalyst for the synthesis of unsymmetrical diorganyl selanes. Heteroaryl iodides, including thiophene and pyridine derivatives, were very tolerant in their reactions. Yasuike et al. (contribution 12) explored the regioselective C–H selenation of indoles with diaryl diselenides for the general synthesis of 3-selanylindoles by using BiI_3_ as a catalyst. Their reaction displayed excellent atomic economy through the utilization of both selanyl groups of the diselenides. Lhoták et al. (contribution 13) reported the Sonogashira coupling of *meta*-iodocalix[4]arene with terminal acetylenes to obtain the corresponding alkynylcalixarenes, which were subsequently subjected to FeCl_3_- and diorganyl diselenide-mediated electrophilic closure. They found that the calix[4]arenes provided totally different bridging products than the non-macrocyclic starting materials. Blažević et al. (contribution 14) described using morphological changes and changes in glucosinolate content in broccoli and rocket to track the stress caused by selenate. Lukesh et al. (contribution 15) highlighted new H_2_Se-releasing chemical tools and methods for selenide detection and quantification. In their review, they also discussed perspectives and unsolved problems related to H_2_Se donation and detection. 

I hope that this Special Issue will be useful to scientists, researchers, and graduate students in the fields of organic chemistry, material chemistry, chemical biology, and computational chemistry, and thank my many prominent colleagues for their contributions. 
